# Biodiversity Patterns and Community Construction in Subtropical Forests Driven by Species Phylogenetic Environments

**DOI:** 10.3390/plants14152397

**Published:** 2025-08-02

**Authors:** Pengcheng Liu, Jiejie Jiao, Chuping Wu, Weizhong Shao, Xuesong Liu, Liangjin Yao

**Affiliations:** 1 Zhejiang Academy of Forestry, Hangzhou 310023, China; lpc1350210@163.com (P.L.); jjjjust@163.com (J.J.); wcp1117@hotmail.com (C.W.); 2 Forestry College, Beihua University, Jilin 132013, China; 3 Jiande Forestry Bureau, Jiande 311600, China; shaoyunzhu@126.com (W.S.); 13968125826@163.com (X.L.)

**Keywords:** species diversity, phylogenetic diversity, soil factors, community assembly

## Abstract

To explore the characteristics of species diversity and phylogenetic diversity, as well as the dominant processes of community construction, in different forest types (deciduous broad-leaved forest, mixed coniferous and broad-leaved forest, and Chinese fir plantation) in subtropical regions, analyze the specific driving patterns of soil nutrients and other environmental factors on the formation of forest diversity in different forest types, and clarify the differences in response to environmental heterogeneity between natural forests and plantation forests. Based on 48 fixed monitoring plots of 50 m × 50 m in Shouchang Forest Farm, Jiande City, Zhejiang Province, woody plants with a diameter at breast height ≥5 cm were investigated. Species diversity indices (Margalef index, Shannon–Wiener index, Simpson index, and Pielou index), phylogenetic structure index (PD), and environmental factors were used to analyze the relationship between diversity characteristics and environmental factors through variance analysis, correlation analysis, and generalized linear models. Phylogenetic structural indices (NRI and NTI) were used, combined with a random zero model, to explore the mechanisms of community construction in different forest types. Research has found that (1) the deciduous broad-leaved forest had the highest species diversity (Margalef index of 4.121 ± 1.425) and phylogenetic diversity (PD index of 21.265 ± 7.796), significantly higher than the mixed coniferous and broad-leaved forest and the Chinese fir plantation (*p* < 0.05); (2) there is a significant positive correlation between species richness and phylogenetic diversity, with the best fit being AIC = 70.5636 and R^2^ = 0.9419 in broad-leaved forests; however, the contribution of evenness is limited; (3) the specific effects of soil factors on different forest types: available phosphorus (AP) is negatively correlated with the diversity of deciduous broad-leaved forests (*p* < 0.05), total phosphorus (TP) promotes the diversity of coniferous and broad-leaved mixed forests, while the diversity of Chinese fir plantations is significantly negatively correlated with total nitrogen (TN); (4) the phylogenetic structure of three different forest types shows a divergent pattern in deciduous broad-leaved forests, indicating that competition and exclusion dominate the construction of deciduous broad-leaved forests; the aggregation mode of Chinese fir plantation indicates that environmental filtering dominates the construction of Chinese fir plantation; the mixed coniferous and broad-leaved forest is a transitional model, indicating that the mixed coniferous and broad-leaved forest is influenced by both stochastic processes and ecological niche processes. In different forest types in subtropical regions, the species and phylogenetic diversity of broad-leaved forests is significantly higher than in other forest types. The impact of soil nutrients on the diversity of different forest types varies, and the characteristics of community construction in different forest types are also different. This indicates the importance of protecting the original vegetation and provides a scientific basis for improving the ecological function of artificial forest ecosystems through structural adjustment. The research results have important practical guidance value for sustainable forest management and biodiversity conservation in the region.

## 1. Introduction

In recent years, global climate change has intensified [1], posing a serious challenge to biodiversity. Species adapt through evolution or migration to cope with environmental changes, and some species face the risk of extinction due to their inability to adapt. Based on species distribution models [2], it is predicted that, by 2025, 70.45% of benzoin species in China’s subtropical evergreen broad-leaved forest areas will experience a decrease in their suitable habitats, while only 29.55% will experience an increase. This indicates that climate change is causing significant changes in the suitable habitats of some species—the distribution range of some species is expanding, while the suitable habitats of more species are showing a decreasing trend. Ecosystem stability depends on community structure. Communities with species that occupy diverse ecological niches can better sustain ecosystem functions, aiding in maintenance and restoration. Different species exhibit differential responses to these changes [3,4]. In the study by Tavankar et al. [5], it was found that protected stands under different management methods in natural forests have richer species diversity than those restored after 25 years of selective thinning, which is beneficial for the stability and restoration of ecosystems. In the assessment of ecosystem stability, the species richness index is widely used, and multiple studies have shown that ecosystems with higher species richness usually have stronger stability [4,6,7,8]. Maestre et al. [9] found through comprehensive analysis of ecosystems in 224 arid regions worldwide that although annual average temperature and soil sand content are the strongest predictors of ecosystem multifunctionality, the optimal models selected based on the AIC criterion (explanatory power > 55%) all contain the key variable of species richness, indicating its irreplaceable role in maintaining ecosystem function. However, species richness can only explain a small part of ecosystem functional variation and cannot reflect functional differences or evolutionary history between species [10].

Phylogenetic diversity measures the evolutionary relationships among species in a community [11,12]. Through phylogenetic diversity, it is known that when environmental filtering plays a dominant role, community species exhibit clustered distribution, representing the composition of closely related species in the community. When competition and exclusion play a dominant role, community species tend to disperse and have distant phylogenetic relationships [13]. It is worth noting that phylogenetic signal analysis can distinguish between adaptive evolution and neutral evolution processes, which has important scientific value for understanding the maintenance mechanisms of biodiversity [14,15]. Research has found that phylogenetic diversity is more explanatory of changes in community productivity than species richness or functional diversity [16,17]; however, using only phylogenetic diversity to assess ecosystems ignores information on community diversity in species evolution.

Therefore, phylogenetic diversity reflects the historical constraints of community construction through evolutionary relationships between species, such as total branch length [12], while species diversity indicates the current utilization status of ecological niches. The combination of the two can distinguish between neutral processes and niche processes, revealing the historical process of community construction. This approach helps to understand how species, communities, and ecosystems are related through evolutionary time. In community construction research, the Net Lineage Relationship Index (NRI) and Net Nearest Species Relationship Index (NTI) are core tools for distinguishing the aggregation or dispersion of phylogenetic structures [18]. The former quantifies the overall phylogenetic relationships of species within a community and reflects their similarity, while the latter focuses on the phylogenetic distance between species and their closest relatives and reflects their mutual influence. Taking the study of karst and non-karst mountain forests in Guangxi by Zeng et al. [19] as an example, they found that NRI and NTI exhibited aggregation in all three major habitats, indicating that environmental filtering dominated community construction and promoted the aggregation of closely related species due to shared adaptive traits. Furthermore, when both NRI and NTI are greater than 0, the phylogenetic structure aggregates, the community is dominated by closely related species, and environmental filtering dominates. When both NRI and NTI are less than 0, the phylogenetic structure diverges, and the community is dominated by distant species, with competition and exclusion dominating [18,20].

Environmental heterogeneity has a significant impact on the construction of phylogenetic diversity, exhibiting complex regional differences along altitude gradients. Research on Mount Kenya [21] has shown that the phylogenetic structures of both low mountain wet forests and high mountain forests tend to be dispersed. However, Li et al. [22] found that the low altitude areas exhibit a divergent structure, while the high altitude areas exhibit a clustered structure. Huang et al. [23] found that the high-altitude areas exhibit divergent and low-altitude clustered structures in Gutian Mountain, revealing significant differences in the role of altitude in community construction due to geographical background and species composition. In terms of soil factors, research on tropical seasonal rainforests in Xishuangbanna [24], China, has confirmed that phosphorus is the core driving factor affecting the phylogenetic structure of trees, while nitrogen has not shown significant effects. These studies demonstrate the complex regulation of environmental factors on community building mechanisms from multidimensional perspectives such as altitude gradients and soil nutrients.

Jiande belongs to Hangzhou City, located in the western part of Zhejiang Province, upstream of the Qiantang River. It belongs to the subtropical monsoon climate zone and is rich in forest resources, mainly including broad-leaved forests, coniferous and broad-leaved mixed forests, Chinese fir plantations, etc. At present, research mainly focuses on biodiversity and genetic diversity, such as the diversity of understory vegetation after nurturing and thinning, and the genetic diversity of *Machilus pauhoi* Kaneh. (Lauraceae) [25,26]. There has been no study on the effects of phylogenetic diversity and soil factors on its distribution. In view of this, it is currently unclear what the patterns of species diversity and phylogenetic diversity are among different subtropical forest types, as well as the community assembly mechanisms connecting them with soil factors. Taking the distribution of different forest types in Shouchang Forest Farm, Jiande City, as the research object, the main research objectives are as follows: (1) To evaluate the relationship between species diversity and phylogenetic diversity in deciduous broad-leaved forests, coniferous–broadleaved mixed forests and Chinese fir plantations; (2) To determine the effects of soil factors on the diversity characteristics of different forest types; (3) To identify the dominant community assembly mechanisms (environmental filtering, competitive exclusion or random processes) of each forest type through phylogenetic structure indices (NRI/NTI). By exploring the species diversity and phylogenetic diversity of different forest types in the intertidal forest area of Shouchang Forest Farm in Jiande City, as well as the impact of soil factors on changes in different forest types, this study provides certain scientific value for community construction and understanding of the maintenance mechanism of biodiversity, and provides a reference for protecting species diversity.

## 2. Results

### 2.1. Distribution Characteristics of Species and Phylogenetic Diversity in Different Forest Types

Based on Objective 1, the evaluation of the relationship between species diversity and phylogenetic diversity in deciduous broad-leaved forests, coniferous–broadleaved mixed forests, and Chinese fir plantations is shown in Table 1. Different forest types have a significant impact on the species diversity of species communities, with the highest Margalef of 4.121 in deciduous broad-leaved forests and the lowest of 2.049 in Chinese fir plantations. The phylogenetic diversity index (PD) is the highest in broad-leaved forests, at 21.265, and the lowest in Chinese fir plantations, at 10.034. The Simpson index, Shannon–Wiener index, and Pielou index of three different forest types all increase with the increase in species numbers.

### 2.2. The Relationship Between Species Diversity and Phylogenetic Diversity of Different Forest Types and the Impact of Environmental Factors on Diversity

**Figure 1:**
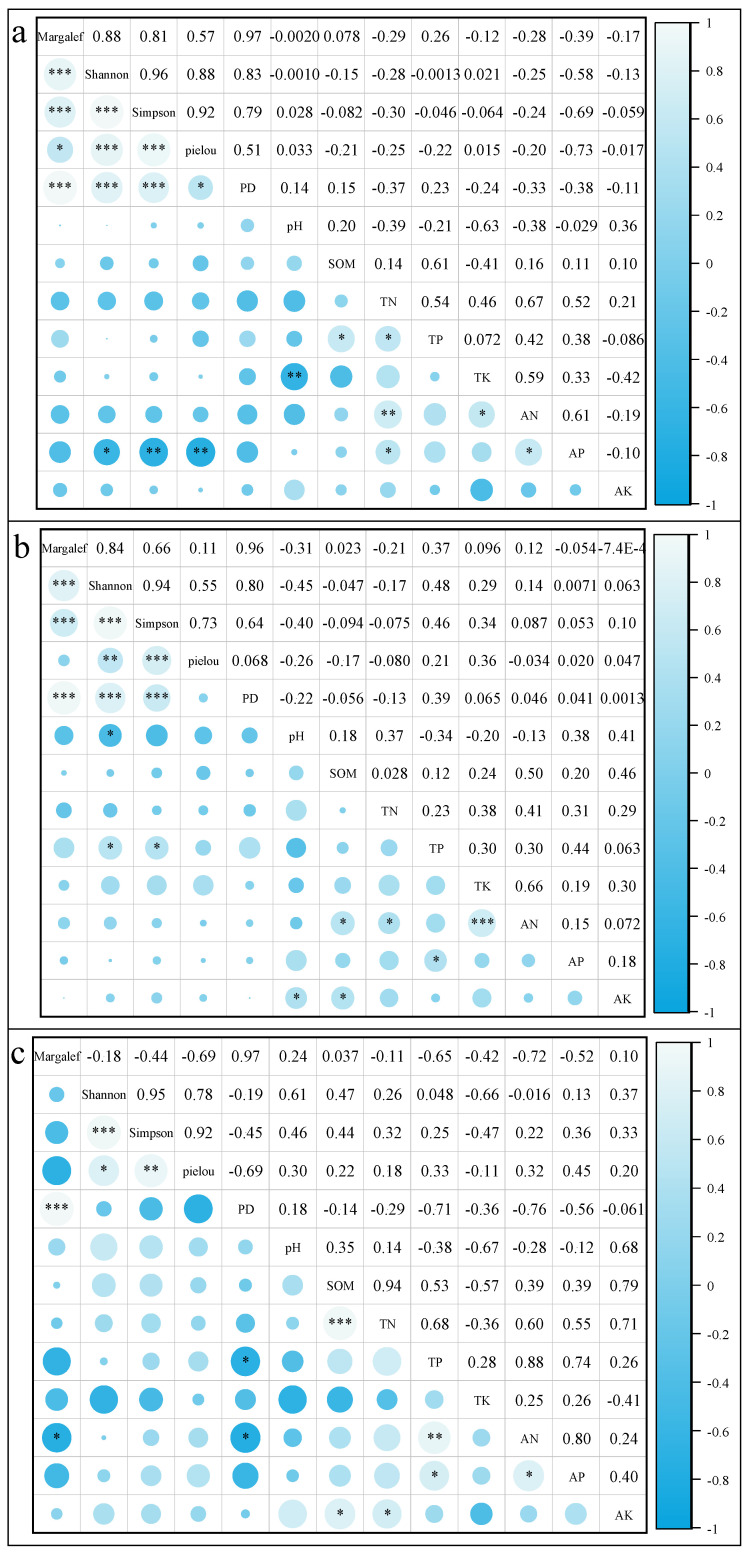
Correlation between species diversity and phylogenetic diversity of different forest types. Note: (**a**), (**b**), and (**c**) represent the correlation between species diversity and structural diversity in deciduous broad-leaved forests, mixed coniferous and broad-leaved forests, and Chinese fir plantations, respectively. * *p* < 0.05, ** *p* < 0.01, and *** *p* < 0.001. SOM = soil organic matter, TN = total nitrogen, TP = total phosphorus, TK = total potassium, AN = alkaline nitrogen, AP = available phosphorus, AK = available potassium.

Based on Objective 2, which involves determining the influence of soil factors on the diversity characteristics of different forest types, it was found that there were significant correlations between the species diversity and phylogenetic diversity indices of the three forest types (Figure 1). The phylogenetic diversity index (PD) in deciduous broad-leaved forests is significantly correlated with the Margalef index, Shannon–Wiener index, and Simpson index (*p* < 0.001), with the highest correlation coefficient of 0.9705 with the Margalef index and a significant correlation with the Pielou index (*p* < 0.05). The phylogenetic diversity index (PD) in mixed coniferous and broad-leaved forests is significantly correlated with the Margalef index, Shannon–Wiener index, and Simpson index (*p* < 0.001), with the highest correlation coefficient of 0.9588 with the Margalef index and no significant correlation with the Pielou index. The phylogenetic diversity index (PD) in Chinese fir plantations is significantly correlated with the Margalef index (*p* < 0.001), with the highest correlation coefficient of 0.9651. There is no significant correlation with the Shannon–Wiener index, Simpson index, and Pielou index.

Univariate regression analysis was conducted on the significant relationships among three different forest types. The phylogenetic diversity index (PD) of the three different forest types showed the strongest linear relationship with the Margalef index, with an AIC of 70.5636 and R^2^ of 0.9419 in the deciduous broad-leaved forest. The AIC in the mixed coniferous and broad-leaved forest is 105.9070, with an R^2^ of 0.9193, while in the Chinese fir plantation, the AIC is 30.6551, with an R^2^ of 0.9314. The PD index increases with the increase in the species richness Margalef index. In deciduous broad-leaved forests, the phylogenetic diversity index (PD) correlates well with the Shannon–Wiener, Simpson, and Pielou indices; however, this relationship is weaker in mixed coniferous–broadleaved forests and Chinese fir plantations (Table 2).

The correlation between species diversity and phylogenetic diversity index (PD) and soil factors varies among three different forest types (Figure 1). In deciduous broad-leaved forests, the Shannon–Wiener index showed a significant negative correlation (*p* < 0.05) with available potassium (AP), while the Simpson index and Pielou index showed a highly significant negative correlation (*p* < 0.01) with available potassium (AP). In mixed coniferous and broad-leaved forests, the Shannon–Wiener index and Simpson index were significantly positively correlated with total phosphorus (TP) (*p* < 0.05), while the Shannon–Wiener index was significantly negatively correlated with pH (*p* < 0.05). In Chinese fir plantations, the Margalef index was significantly negatively correlated with available nitrogen (AN) (*p* < 0.05), while the PD index was significantly negatively correlated with total nitrogen (TN) and available nitrogen (AN) (*p* < 0.05).

A generalized linear model was used to fit the significant relationship between community indices and soil factors for three different forest types (Table 3). The Simpson index and available potassium (AP) showed the best fitting effect in deciduous broad-leaved forests, with an AIC of −7.664. The Simpson index has the best fitting effect on total phosphorus (TP) in mixed coniferous and broad-leaved forests, with an AIC of −16.265. In Chinese fir plantations, the Margalef index has the best fitting effect on alkaline nitrogen (AN), with an AIC of 12.780.

### 2.3. Community Construction and Driving Mechanisms of Different Forest Types

**Figure 2:**
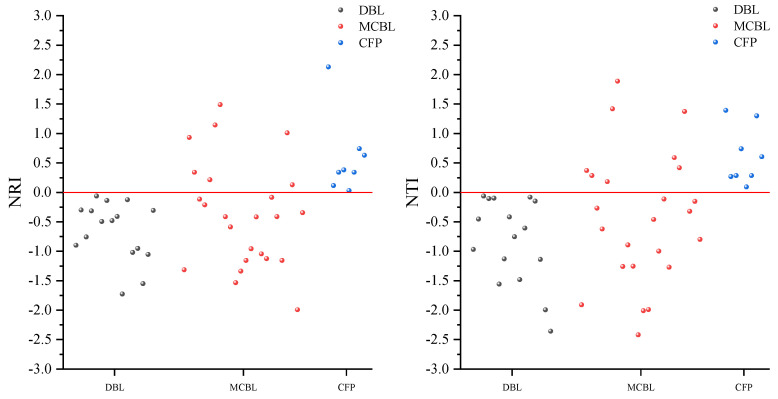
Distribution characteristics of phylogenetic structures of different forest types. Note: DBL stands for deciduous broad-leaved forest, MCBL stands for mixed coniferous and broad-leaved forest, and CFP stands for Chinese fir planning.

Based on Objective 3, the dominant community assembly mechanisms of each forest type were identified through phylogenetic structure indices (NRI/NTI). It was found that the phylogenetic structure indices NRI and NTI of the three different forest types showed differentiated characteristics (Figure 2), reflecting different community construction mechanisms: in deciduous broad-leaved forests, NRI and NTI are both less than 0, indicating a lower community structure than in a random state. This phenomenon indicates that interspecific competition within the community drives phylogenetic dispersion, and species coexist through niche differentiation. Different species occupy unique ecological niches to reduce resource competition and maintain community stability. In Chinese fir plantations, both NRI and NTI are greater than 0, indicating a higher community structure than in a random state. The construction process is mainly influenced by environmental filtering, leading to phylogenetic clustering. The aggregation of closely related species due to shared similar environmental needs reflects the dominant role of environmental filtering in species composition. In the mixed coniferous and broad-leaved forest, NRI and NTI showed a trend from less than 0 to greater than 0, indicating that there are both parts of the community structure that are higher than or lower than the random state. This indicates that the community construction mechanism is more complex and is simultaneously affected by both competitive exclusion and environmental filtering, resulting in a coexistence of dispersed and aggregated phylogenetic patterns, reflecting the combined effects of stochastic processes and niche processes.

## 3. Discussion

### 3.1. Distribution Characteristics of Species and Phylogenetic Diversity

This study found that the species diversity and phylogenetic diversity (PD) of deciduous broad-leaved forests were significantly higher than those of coniferous broad-leaved mixed forests and Chinese fir plantations. This phenomenon may be related to the complex structure and wider ecological niche utilization of broad-leaved forests. Deciduous broad-leaved forests typically have higher tree species diversity and hierarchical structure, providing more ecological niche space and supporting the coexistence of more species [27]. Phylogenetic diversity (PD) is significantly positively correlated with species richness (Margalef index), indicating that as the number of species increases, the evolutionary history of the community also expands. This result is consistent with existing research [28], further demonstrating the synergistic relationship between species richness and phylogenetic diversity. The study by Li et al. [29] on the Dulong River Valley showed that the species richness and phylogenetic diversity of evergreen broad-leaved forests were significantly higher than those of coniferous forests. This finding is consistent with the highest diversity of broad-leaved forests in this study, indicating that complex community structures may promote species coexistence and accumulation of evolutionary history by providing a wider ecological niche space.

However, the correlation between Pielou’s evenness index and phylogenetic diversity (PD) is weak in all mixed coniferous and broad-leaved forests and Chinese fir, indicating that the contribution of species abundance distribution to phylogenetic diversity is limited. These results support Faith’s theoretical prediction [12] that species richness is the main determinant of phylogenetic diversity, which is consistent with the research findings of Chen et al. [30] on the relationship between species diversity and phylogenetic diversity of forest window plants. This may be because evenness mainly reflects the distribution pattern of individual numbers within a community, while PD relies more on the evolutionary relationships between species. Therefore, when evaluating ecosystem functions, complementary information on species diversity and phylogenetic diversity needs to be considered simultaneously.

### 3.2. Characteristics of Community Construction in Different Forest Types

This study found that the phylogenetic indices (NRI and NTI) were both less than 0 in deciduous broad-leaved forests, indicating that the phylogenetic relationships between species within the community were significantly distant from the random expected level, exhibiting a typical phylogenetic dispersal pattern. This finding supports the hypothesis that competitive exclusion drives community assembly in these forests. It is worth noting that the conclusion of this study is different from the research results of Lu et al. [31] on the clustering of the phylogenetic structure of low altitude monsoon evergreen broad-leaved forests in Ailao Mountain, but consistent with the research conclusion of Li et al. [29] on low altitude broad-leaved forests in this study, coniferous forests showed a significant pattern of phylogenetic structure index (NRI > 0), while mixed coniferous and broad-leaved forests exhibited characteristics of phylogenetic structure that differed from single forest types due to the coexistence of coniferous and broad-leaved tree species. This comparison further reveals that the competitive exclusion-driven phylogenetic dispersion pattern is more common in low-altitude, broad-leaved forests under natural conditions with less human interference. This pattern is not unique to China but has been observed in subtropical ecosystems globally. For instance, studies on the Cerrado subtropical savanna in Brazil have demonstrated that dominant species there exhibit significant phylogenetic overdispersion, primarily driven by competitive exclusion [32]. Similarly, research on subtropical oak communities in Florida, USA, has shown that coexisting oak species have significantly lower phylogenetic relatedness than random expectations, with reduced niche overlap among species within the same clade, further confirming that competitive exclusion shapes the phylogenetic structure of broad-leaved communities [33]. Meanwhile, coniferous forests are more likely to form an environmental filtering-dominated phylogenetic clustering pattern due to their convergent adaptation to specific environmental conditions. This is consistent with the results of this study, where the phylogenetic structural indices (NRI and NTI) of the Chinese fir plantation were both greater than 0, and aligns with the finding of Li et al. [29] that coniferous forests have NRI > 0. This ecological pattern has also been validated in subtropical regions of the Americas. A study on the subtropical elevational gradient in Durango, Mexico, revealed that plant communities in high-altitude areas exhibit significant phylogenetic clustering, and the intensity of clustering increases with the increasing dominance of gymnosperms, particularly *pinus* species. This indicates that environmental filtering is a common driver of the phylogenetic structure of coniferous forests in subtropical regions worldwide [34]. The phylogenetic indices (NRI and NTI) of the mixed coniferous and broad-leaved forest exhibit transitional characteristics of positive and negative alternation, which fully reflect the transitional ecological features of the community. This reflects the dynamic balance mechanism formed by the long-term coexistence of coniferous and broad-leaved tree species through niche differentiation and interspecific interactions, which is different from a single functional group—it is neither completely driven by competition and exclusion dispersion, nor aggregation caused by a single environmental filter, but a transitional manifestation of the interaction between the two ecological processes, forming a mixed mode that combines competition and selection. This discovery provides a new perspective for understanding the response of forest communities to environmental changes, namely that artificial intervention may alter the dominant mechanisms of community construction by regulating interspecific relationships, thereby affecting the maintenance of biodiversity.

### 3.3. The Impact of Environment on Diversity and the Driving Mechanism of Communities

This study found significant differences in the impact of soil nutrients on the diversity patterns of different forest types. In deciduous broad-leaved forests, available phosphorus (AP) is significantly negatively correlated with diversity index (*p* < 0.05), which is consistent with previous research results: phosphorus enrichment may promote the growth of dominant species while inhibiting other species, leading to increased competitive exclusion [35,36]. In mixed coniferous and broad-leaved forests, total phosphorus (TP) is positively correlated with the diversity index, indicating that phosphorus is a key limiting factor for subtropical forest productivity. Increasing its content can alleviate resource constraints, promote the coexistence of coniferous and broad-leaved tree species, and thereby enhance community diversity [37]. Nitrogen’s effects are twofold: moderate levels support species richness, while excess nitrogen can acidify soils, hinder species adaptation, and intensify competition [38,39,40]. In Chinese fir plantations, alkaline nitrogen (AN) is significantly negatively correlated with Margalef index and PD (*p* < 0.05), which may be due to the efficient utilization of nitrogen by Chinese fir as a single dominant species inhibiting the growth of other species [41], further confirming that nutrient enrichment may alter the strength and direction of interspecific relationships [42]. The above results indicate that soil factors have specific effects on the diversity patterns of different forest types through the interaction of “environmental filtering resource competition”: in natural forests, soil nutrients affect diversity by regulating the intensity of interspecific competition. In Chinese fir plantations, nutrient content mainly enhances the environmental adaptation advantage of dominant species and intensifies the environmental filtering effect.

## 4. Materials and Methods

### 4.1. Overview of the Study Area

Shouchang Forest Farm in Jiande City is located in Shouchang Town, Jiande City (119.28° E, 29.47° N). It belongs to a subtropical monsoon climate, warm and humid, with four distinct seasons. The annual average temperature is 17.8 °C, with extreme high temperatures of 38 °C and extreme low temperatures of −6 °C. The average annual precipitation is between 1500 and 1800 mm, concentrated from April to June, with an average relative humidity of 82%. The annual sunshine hours are 1762 h, and the frost-free period is 265 days throughout the year. The forest farm is dominated by low mountain and hilly landforms, with an altitude range of 200–700 m and a slope mostly between 25 and 45 degrees. Soil type: Mainly yellow soil, with some areas being erosive red soil. The soil layer thickness is between 30 and 80 cm, and the soil layer is deep and fertile, suitable for the growth of various subtropical plants. Shouchang Forest Farm is rich in species and diverse in vegetation types. The main tree species include *Sassafras tzumu* (Hemsl.) Hemsl. (Lauraceae), *Liriodendron chinense* (Hemsl.) Sarg. (Magnoliaceae), *Dalbergia hupeana* Hance (Fabaceae), *Triadica sebifera* (L.) Small (Euphorbiaceae), *Liquidambar formosana* Hance (Hamamelidaceae), *Pinus massoniana* Lamb. (Pinaceae), and *Cunninghamia lanceolata* (Lamb.) Hook. (Cupressaceae).

### 4.2. Experimental Design and Data Acquisition

#### 4.2.1. Sample Plot Setting and Vegetation Survey

Based on the CTFS sampling standards, 48 fixed monitoring plots of 50 m × 50 m were established in the Tanxia Forest Area of Shouchang Forest Farm in Jiande City. Plants with a diameter at breast height (DBH) ≥ 5 cm were surveyed, and information such as plot, tree number, tree species, DBH, tree height, and crown width was recorded. Species names were checked against the species list in Flora of China Online (https://www.iplant.cn/frps).

#### 4.2.2. Forest Type Classification

Plots were classified based on the Importance Values Index (IVI) of tree species within them [43]. The formula for calculating the Importance Values Index is IVI = 100 × (Relative Density + Relative Frequency + Relative Basal Area)/3. A plot was classified as a Chinese fir plantation (with a total of 8 plots) if the Importance Values Index (IVI) of Chinese fir in the plot was greater than 66.7%, indicating that Chinese fir was the absolute dominant species. It was categorized as a coniferous–broadleaved mixed forest (with a total of 24 plots) when the Importance Values Index (IVI) of Chinese fir was between 33.3% and 66.7%, meaning that Chinese fir and broad-leaved tree species co-dominated the plot. A plot was defined as a deciduous broad-leaved forest (with a total of 16 plots) if the Importance Values Index (IVI) of Chinese fir was less than 33% (i.e., the total importance value of broad-leaved tree species was more than 66.7%), showing that broad-leaved tree species were the absolute dominant species (Table 4).

### 4.3. Soil Factor Investigation

#### 4.3.1. Soil Sampling

Randomly select 5 sampling points within each plot, first remove impurities such as stones and debris from the soil surface, and then use a soil drill to collect soil samples at a depth of 0–20 cm at each sampling point. After mixing the soil samples from 5 sampling points evenly, a “quartering method” was used to take 1000 g of soil sample as the soil index measurement sample for the site.

#### 4.3.2. Soil Determination

There are a total of eight soil indicators measured this time, including pH, soil organic matter content (SOM, g·kg^−1^), total nitrogen (TN, g·kg^−1^), total phosphorus (TP, g·kg^−1^), total potassium (TK, g·kg^−1^), alkaline nitrogen (AN, mg·kg^−1^), available phosphorus (AP, mg·kg^−1^), and available potassium (AK, mg·kg^−1^). The total nitrogen content of soil is determined using an elemental analyzer [44], while the alkaline hydrolysis nitrogen content is determined using the alkaline hydrolysis diffusion method [45]. The determination of total phosphorus and total potassium content is carried out using a plasma sensing coupling device [22], the determination of effective phosphorus content is carried out using sodium bicarbonate leaching, molybdenum antimony anti spectrophotometric method [45], and the determination of available potassium content is carried out using ammonium acetate leaching flame photometer method [45]. The organic matter content is determined using the potassium dichromate oxidation external heating method [45], and the pH content is determined using the electrode method [22].

### 4.4. Species Diversity Calculation

Select Margalef, Simpson, Shannon–Wiener, and Pielou indices as species diversity indices [46,47].

The formula for calculating the Margalef index is as follows:

$$D=\frac{S-1}{ln\;N}$$

*N*: total number of individuals, *S*: total number of species.

The Simpson index calculation formula is as follows:

$$D=-ln\left[\sum_{i=1}^{s}{\left(\frac{N_{i}}{N}\right)}^{2}\right]$$

*N*: total number of individuals, *N_i_* is the number of individuals of the *i*-th species.

The calculation formula for the Shannon–Wiener index is as follows:

$$H=-\sum_{i=1}^{S}p_{i}\;ln(p_{i})$$

*S*: total number of species, *p_i_:* represents the abundance ratio of the *i*-th species.

The calculation formula for the Pielou index is as follows:

J=H/ln S

*H*: Shannon–Wiener index, *S*: total number of species.

### 4.5. Phylogenetic Diversity Calculation

Using the plant lineage library software Phylomatic V3.0, a phylogenetic tree of the angiosperm classification system APG III was generated. Based on this, the Picante software package in R was used to calculate the phylogenetic diversity and structure [11]. The phylogenetic diversity index was PD (Phytogenetic diversity), which is the sum of the lengths of all species connected to the phylogenetic tree.

### 4.6. Calculation of Phylogenetic Structure Index

Quantify the phylogenetic structure index using the Net Kinship Index (NRI) and Net Nearest Taxa Index (NTI) [18]. Construct a random community using the zero model and compare the phylogenetic patterns of the actual community with those of the random community.

The calculation formulas for NIR and NTI are as follows:

$$\mathrm{NRI}=-1\times \frac{\mathrm{MPDs}-\mathrm{MPDmds}}{\mathrm{SD}(\mathrm{MPDmds})}$$

$$\mathrm{NTI}=-1\times \frac{\mathrm{MNTDs}-\mathrm{MNTDmds}}{\mathrm{SD}(\mathrm{MPDmds})}$$

MPDS is the average spectral distance observation value. MNTDS is the average observation value of the distance between the nearest neighboring lineages. MPDmds and MNTDmds represent the average and nearest neighbor lineage distances simulated after 999 random selections, with SD as the standard deviation.

### 4.7. Data Analysis

Using one-way analysis of variance to calculate differences in species diversity, as well as differences in phylogenetic diversity (PD) and phylogenetic structural indices (NRI and NTI). For normally distributed data, Pearson correlation analysis is used to examine the relationship between species diversity and phylogenetic diversity (PD), as well as phylogenetic structure (NRI and NTI). Then, univariate linear regression analysis is used to fit species diversity and phylogenetic diversity to determine if there is a linear relationship. The Akaike Information Criterion (AIC) and R^2^ are used to evaluate the fitting effect.

Use Pearson correlation analysis to investigate the relationship between soil factors, species diversity, and phylogenetic diversity. For factors with strong significance, the Gaussian model based on normal distribution in the generalized linear model is used to explore the driving factors that affect the phylogenetic index and species diversity index. The Akaike Information Criterion (AIC) is used to evaluate the fitting effect, and variables with a variance inflation factor (VIF) greater than 10 are excluded from the regression model. The model data are then reselected for fitting to reduce collinearity and obtain the optimal model of soil factors for species diversity, phylogenetic diversity, and system structure.

The calculation of importance values was completed in Excel 2016, and one-way analysis of variance was performed in SPSS 27. Species diversity analysis was conducted using the vegan package [48], phylogenetic analysis using the picante package [11,29], correlation analysis using the corrplot package [22], and linear model construction using the broom package [22], all of which were implemented in R version 4.4.2 [49]. Graphs were plotted using Origin 2021.

## 5. Conclusions

This study analyzed the diversity distribution characteristics, environmental factor driving forces, and community assembly mechanisms of three different forest types. The main conclusions are as follows: (1) Diversity distribution characteristics: The Margalef index and phylogenetic diversity of deciduous broad-leaved forests were the highest. Chinese fir plantations had the lowest Margalef index and phylogenetic diversity due to their single dominant structure. The Margalef index was the best indicator for predicting phylogenetic diversity, reflecting that an increase in species quantity may be accompanied by the expansion of evolutionary lineages. However, the Pielou index made limited contributions, indicating that the evenness of species distribution within the community was not a key factor affecting differences in evolutionary history. (2) Environmental driving factors: Soil nutrients had differentiated impacts on the diversity patterns of different forest types. In deciduous broad-leaved forests, AP was negatively correlated with diversity, which might be attributed to intensified competitive exclusion caused by phosphorus enrichment. In coniferous–broadleaved mixed forests, TP showed a positive correlation with diversity, suggesting that a moderate increase in phosphorus could promote species coexistence. In Chinese fir plantations, AN was negatively correlated with diversity, reflecting that the efficient utilization of nitrogen by the single dominant species inhibited other species. (3) Community assembly mechanisms: Regarding the phylogenetic structures of the three different forest types, deciduous broad-leaved forests showed a divergent pattern, indicating that competitive exclusion dominated their assembly. Chinese fir plantations presented an aggregated pattern, suggesting that environmental filtering played a leading role in their assembly. Coniferous–broadleaved mixed forests exhibited a transitional pattern, indicating that they were affected by the combined mechanisms of stochastic processes and niche processes. Protecting subtropical deciduous broad-leaved forests is crucial for preserving regional biodiversity and ecosystem functions. In the practice of protection, high—intensity human disturbances (such as commercial logging and forest land reclamation) need to be restricted. In forest management, phosphate fertilizer use should be limited to prevent excessive phosphorus from exacerbating competitive exclusion. Regarding the ecological vulnerability problem brought by the single—planting pattern of Chinese fir plantations, it is recommended to adopt comprehensive transformation measures. Firstly, thin the forest to reduce the stand density, so as to improve the light and space resource conditions in the forest, and create a suitable growth environment for the introduction of other tree species. Secondly, select and replant native broad-leaved tree species suitable for growth, such as Fagaceae and Lauraceae, to ensure that the mixed-planting proportion reaches more than 30%, so as to optimize the vertical structure of the community and enhance the functional diversity. Appropriately add phosphate fertilizer to improve the soil nutrient balance, create favorable conditions for the successful settlement and healthy growth of the mixed-planting tree species, and, finally, achieve the coordinated improvement of species diversity and community stability in the plantation ecosystem. This study offers insights into community assembly. Future research could combine functional trait analysis and long-term monitoring to explore biodiversity maintenance under climate change, aiding adaptive forest management.

## Figures and Tables

**Table 1:** Changes in species diversity and phylogenetic diversity of different forest types.

Forest Type	Margalef	Shannon	Simpson	Pielou	PD
Deciduous broad-leaved forest	4.121 ± 1.425 a	1.448 ± 0.632 a	0.630 ± 0.225 a	0.616 ± 0.166 a	21.265 ± 7.796 a
Mixed coniferous and broad-leaved forest	3.507 ± 1.158 a	1.019 ± 0.426 a	0.469 ± 0.175 a	0.501 ± 0.155 ab	18.279 ± 6.975 ab
Chinese fir plantation	2.049 ± 0.570 b	0.377 ± 0.156 b	0.198 ± 0.121 b	0.366 ± 0.248 ab	10.034 ± 4.610 b

Note: Different letters in the same column represent significant differences in forest types (*p* < 0.05).

**Table 2:** Regression analysis of species diversity and phylogenetic index of different forest types.

Forest Type	Phylogenetic Diversity Index	Species Diversity Index	AIC	R^2^	*p*-Value
Deciduous broad-leaved forest	PD	Margalef	70.5636	0.9419	**
	PD	Shannon	97.4848	0.6874	**
	PD	Simpson	100.459	0.6235	**
	PD	Pielou	111.1831	0.2641	*
Mixed coniferous and broad-leaved forest	PD	Margalef	105.9070	0.9193	**
	PD	Shannon	142.2155	0.6337	**
	PD	Simpson	153.6855	0.4093	**
	PD	Pielou	166.2070	0.0047	/
Chinese fir plantation	PD	Margalef	30.6551	0.9314	**
	PD	Shannon	51.7857	0.0368	/
	PD	Simpson	50.2968	0.2004	/
	PD	Pielou	46.8579	0.4798	/

Note: * *p* < 0.05, ** *p* < 0.01.

**Table 3:** Generalized linear model analysis of the relationship between various indices and soil factors.

Different Types of Forests	Index	Soil Factors	Regression Coefficient	Standard Error	*t-*Values	*p*-Value	AIC
Deciduous broad-leaved forest	Shannon	AP	−0.251	0.132	−2.650	*	29.171
	Simpson	AP	−0.149	0.042	−3.551	**	−7.664
	Pielou	AP	−0.116	0.029	−3.969	**	−19.070
Mixed coniferous and broad-leaved forest	Shannon	TP	3.782	1.463	2.586	*	25.811
	Shannon	pH	−0.389	0.165	−2.357	*	26.777
	Simpson	TP	1.491	0.609	2.449	*	−16.265
Chinese fir plantation	Margalef	AN	−0.0123	0.005	−2.548	*	12.780
	PD	AN	−3.998	5.314	−0.752	/	51.364
	PD	TP	−123.77	49.48	−2.501	*	46.371

Note: * *p* < 0.05, ** *p* < 0.01. Linear regression and collinearity diagnostics were performed in SPSS 27. The screened factors were all significant factors, and no VIF (variance inflation factor) was found to be greater than 10.

**Table 4:** The top 5 species in terms of importance values for different forest types.

Forest Type	Species	Importance Values Index (%)
Deciduous broad-leaved forest	*Liquidambar formosana* Hance (Hamamelidaceae)	16.14
	*Cunninghamia lanceolata* (Lamb.) Hook. (Cupressaceae)	13.85
	*Pinus massoniana* Lamb. (Pinaceae)	11.84
	*Sassafras tzumu* (Hemsl.) Hemsl. (Lauraceae)	8.18
	*Cinnamomum japonicum Siebold (Lauraceae)*	7.19
Mixed coniferous and broad-leaved forest	*Cunninghamia lanceolata* (Lamb.) Hook. (Cupressaceae)	35.72
	*Pinus massoniana* Lamb. (Pinaceae)	21.05
	*Sassafras tzumu* (Hemsl.) Hemsl. (Lauraceae)	10.59
	*Liriodendron chinense* (Hemsl.) Sarg. (Magnoliaceae)	8.04
	*Paulownia fortune* *(Seem.) Hemsl. (Paulowniaceae)*	3.56
Chinese fir plantation	*Cunninghamia lanceolata* (Lamb.) Hook. (Cupressaceae)	75.55
	*Sassafras tzumu* (Hemsl.) Hemsl. (Lauraceae)	17.17
	*Pinus massoniana* Lamb. (Pinaceae)	2.60
	*Liriodendron chinense* (Hemsl.) Sarg. (Magnoliaceae)	1.05
	*Dalbergia hupeana* Hance (Fabaceae)	1.04

## Data Availability

The raw data supporting the conclusions of this article will be made available by the authors on request.
